# Producing HIV estimates: from global advocacy to country planning and impact measurement

**DOI:** 10.1080/16549716.2017.1291169

**Published:** 2017-05-22

**Authors:** Mary Mahy, Tim Brown, John Stover, Neff Walker, Karen Stanecki, Wilford Kirungi, Txema Garcia-Calleja, Peter D. Ghys

**Affiliations:** ^a^ Strategic Information and Evaluation Department, UNAIDS, Geneva, Switzerland; ^b^ East-West Center, Honolulu, HI, USA; ^c^ Avenir Health, Glastonbury, CT, USA; ^d^ Department of International Health, Bloomberg School of Public Health, Baltimore, MD, USA; ^e^ Independent Consultant, Washington, DC, USA; ^f^ Ministry of Health, Kampala, Uganda; ^g^ HIV Department, World Health Organization, Geneva, Switzerland

**Keywords:** Bringing the indicators home: Country perspective on the utility of global estimates for health indicators (WHO), Estimation, HIV, models, strategic information

## Abstract

**Background**: The development of global HIV estimates has been critical for understanding, advocating for and funding the HIV response. The process of generating HIV estimates has been cited as the gold standard for public health estimates.

**Objective**: This paper provides important lessons from an international scientific collaboration and provides a useful model for those producing public health estimates in other fields.

**Design**: Through the compilation and review of published journal articles, United Nations reports, other documents and personal experience we compiled historical information about the estimates and identified potential lessons for other public health estimation efforts.

**Results**: Through the development of core partnerships with country teams, implementers, demographers, mathematicians, epidemiologists and international organizations, UNAIDS has led a process to develop the capacity of country teams to produce internationally comparable HIV estimates. The guidance provided by these experts has led to refinements in the estimated numbers of people living with HIV, new HIV infections and AIDS-related deaths over the past 20 years.

A number of important updates to the methods since 1997 resulted in fluctuations in the estimated levels, trends and impact of HIV. The largest correction occurred between the 2005 and 2007 rounds with the additions of household survey data into the models. In 2001 the UNAIDS models at that time estimated there were 40 million people living with HIV. In 2016, improved models estimate there were 30 million (27.6–32.7 million) people living with HIV in 2001.

**Conclusions**: Country ownership of the estimation tools has allowed for additional uses of the results than had the results been produced by researchers or a team in Geneva. Guidance from a reference group and input from country teams have led to critical improvements in the models over time. Those changes have improved countries’ and stakeholders’ understanding of the HIV epidemic.

## Background

Public health responses require evidence to plan, prioritize, implement, monitor, and evaluate public health actions to reduce morbidity and mortality [1–3]. The response to the HIV epidemic has received considerable investment to ensure data and strategic information are available to understand the epidemic [4]. This information is required at the global, regional, country and sub-national levels to focus the response on populations and locations where it is most needed [5].

Due to limited data for many public health responses, modelled estimates are required to provide information where empirical data are not available or insufficient [6–9]. Some simple, logical rules apply to modelled estimates. The data must be of good enough quality to allow program managers at the relevant level to act on the results [1]. The estimates must be available at a frequency that allows timely action to redirect programs. The owners of the estimates should be the public health managers who will act on those estimates [10]. Finally, for global estimates processes the modelled estimates need to be comparable from one country to the next to allow for accurate aggregations and comparisons of the epidemic between countries. Besides use in public health management, HIV estimates are also widely used by funding agencies and donor countries to guide their investments [11,12], and by advocates and civil society to hold those responsible for action to account. In recent years modelled estimates have been created at the sub-national level to identify location-specific programmatic gaps and HIV burden to direct resources to areas of greatest need [13].

The development of HIV estimates by countries around the world has been considered a good model of health estimates processes because of the ownership of the estimates by country teams, the frequency of the estimates and the comparability of the results [14]. This paper explains the evolution of the processes and methods used to develop the HIV estimates and identifies lessons learned related to the estimates process. As indicators linked to the Sustainable Development Goals and related targets are still being defined, we hope the experience from the HIV estimates process will provide useful lessons for other organizations that develop national and global estimates.

## Methods

**Figure 1.**
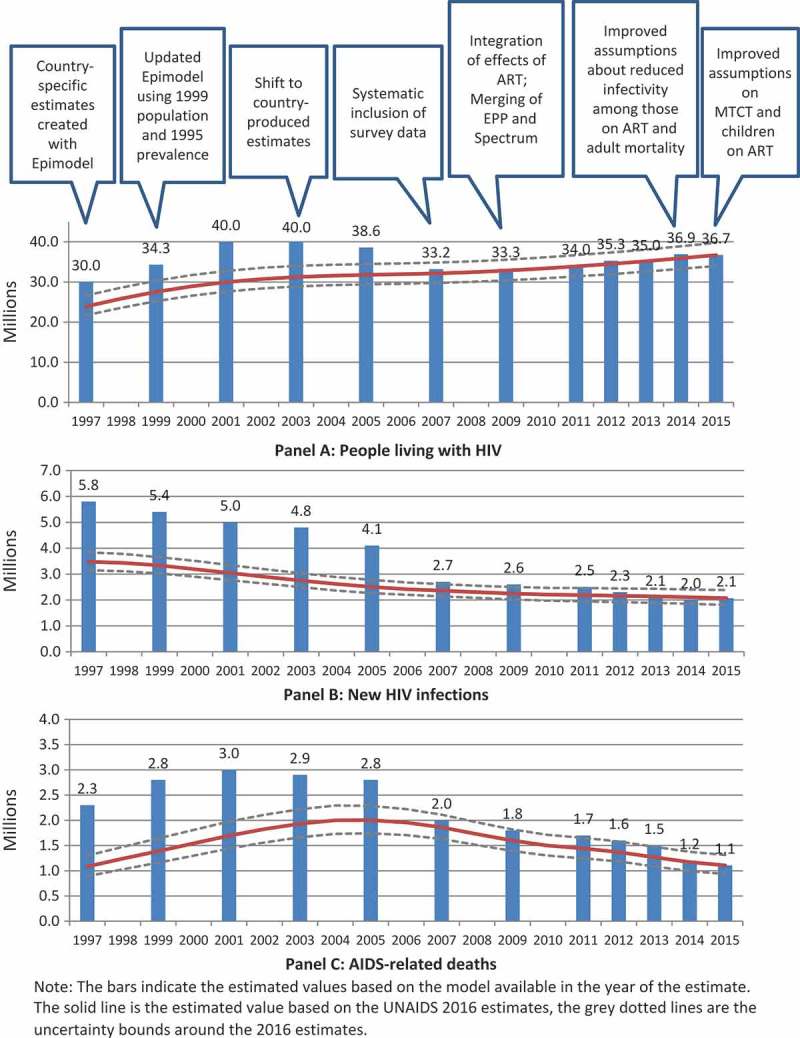
Past and current estimates of the global numbers of people living with HIV, new HIV infections, and AIDS-related deaths and major factors contributing to the changes in past estimates, 1997–2015.

The information compiled for this paper is based on a number of sources. Descriptions of the changes in the methods are based on published manuscripts as well as reports maintained by the UNAIDS Reference Group on Estimates, Modelling and Projections (UNAIDS Reference Group). The biennial journal supplements devoted to the HIV estimates were reviewed to identify major changes and examples of country uses of the estimates. UNAIDS’ archived global reports were used to compile the data included in Figure 1. Finally, the information on the evolving estimation process was obtained from documents within UNAIDS’ internal filing system as well as personal experience – six of the eight authors were involved in the initiation of the UNAIDS HIV estimates process.

## Findings

The findings from this review can be broken down into three parts: the evolution of the process of generating the estimates, refinements to the computational methods, and the impact of these adjustments on the HIV estimates.

### Evolution of the process for generating estimates

#### Initial estimates

The methods for developing the HIV estimates have evolved considerably over the past two decades. In the 1990s the World Health Organization’s Global AIDS Programme developed initial modelling methods and tools [15] and published global, regional and the first set of country estimates of the number of people living with HIV [16]. These and subsequent sets of estimates published by UNAIDS starting in the late 1990s were generated by a small group of individuals based in Geneva, Switzerland, using the available epidemiological data on the HIV epidemic [16,17]. While these estimates were the best available at the time, they were based on small studies which were not representative of the geographic area or populations to which the data were extrapolated. Data were borrowed from data-rich countries to estimate prevalence in data-poor countries. The resulting estimates were used to inform global advocacy campaigns and to understand the relative public health importance of HIV in comparison to other diseases [18].

#### Reference Group on HIV Estimates, Modelling and Projections

Since 1997, a group of experts has been regularly convened to advise UNAIDS on the development of the models used for generating HIV estimates [19]. The UNAIDS Reference Group provides guidance on model structure, on country-specific data to include in the model, and on assumptions and parameters as new scientific data become available. The UNAIDS Reference Group was set up as an ‘open cohort’, allowing the secretariat of this group (initially at Oxford University, later at Imperial College, London) to identify experts and practitioners to join meetings who were best placed to answer the questions at hand. The meeting participants primarily include mathematical modelers, country program managers, monitoring and evaluation specialists, demographers and population cohort study investigators. The results of the reference group discussions are made public through biennial journal supplements [20–25] publishing details about the models’ structure, input data and assumptions as well as through a website that provides summary reports of the meetings of the UNAIDS Reference Group (www.epidem.org).

#### Research partners

Critical participants in the Reference Group meetings have been international research consortiums that provide data used to improve the models. Notably the network for Analysing Longitudinal Population-based HIV/AIDS data on Africa (ALPHA) compiles and analyses data from partners who run demographic and surveillance sites in 10 locations across 6 countries in eastern and southern Africa (http://alpha.lshtm.ac.uk/). Data from this network have influenced numerous parameters [26–32] including the age and sex patterns of incidence and survival among people who did not receive antiretroviral therapy (ART). Similarly important to the evolution of the parameters and assumptions in the model is the International Epidemiologic Databases to Evaluate AIDS (IeDEA) consortium which provides information by age and sex on the survival among people who have received ART including those who have disengaged from care [33]. These international research partners are able to provide complex information on the HIV epidemic that is not available from routine data sources.

#### Building capacity with user-friendly software

Starting in 2003, there was an important shift in the HIV estimates process triggered by the desire for national input. A deliberate effort was made to develop user-friendly computer software and make the estimates methods available to countries. Global capacity building in the use of the models started in 2003 with regional workshops held in Zimbabwe, Benin, Thailand, Guatemala, Jamaica, Croatia, Tunisia and Egypt. Through a well-coordinated effort, UNAIDS and partners developed the capacity of 131 country teams to create estimates using user-friendly software. This transferred ownership of the estimates from Geneva-based technical staff to country teams who needed these data to inform and empower their countries’ politicians, program managers and civil society organizations. Country teams were developed and included surveillance specialists from the Ministry of Health, the National AIDS Coordinating mechanisms, demographers, program officers and country-based staff of development partners. Regional capacity-building workshops have been held every two years since 2003. Additional in-country capacity-building activities have also been conducted, most recently to support the development of sub-national estimates. The continuation of the workshops is necessary due to continuing changes in the models and turnover in the country staff working on the estimates, evolving data sources and systems, and changes in output required to improve national and global processes.

Since 2003 the workshops have been led and coordinated by UNAIDS with substantial support from the model developers (Avenir Health and East-West Center) US Government agencies working on HIV, as well as the World Health Organization (WHO), UNICEF and other interested development partners. Partners provide facilitators for the estimates workshops ensuring a broad understanding of the estimation methods by development partners, and ensuring consistent use of the results and support to countries. Participants are trained on the use and functions of the software as well as on updates since the previous round of estimates.

#### Frequency of producing estimates

There is increasing demand for strategic information to be available on a ‘real-time’ basis. Since 2013 UNAIDS has supported countries to produce estimates on an annual basis, compared to a biennial frequency before 2013. Given the rapid expansion of prevention of mother to child transmission (PMTCT) services and ART coverage, this change in frequency was an important adjustment for measuring the impact of HIV interventions. In the years between the biennial workshops, changes to the software are limited so country teams are able to easily update their previous year’s file and produce a new set of estimates, including historical estimates. Support to country teams is provided by UNAIDS staff and technical partners over email or conference calls, as necessary, to update the estimation files.

#### Quality check on data entered

Another improvement in the HIV estimates is the review of the programmatic ART and PMTCT data entered by country teams in the software through a global validation process. Country program data are compared with other sources (such as the global AIDS reporting processes and partner reporting systems) and over time. Potential discrepancies are identified and discussed with country teams. The final validated program data are used in the models, ensuring internal validity between the countries’ program data and the HIV estimates.

#### Transparency

UNAIDS improved the transparency of the estimates in 2014 by making the country files publicly available over the Internet. UNAIDS seeks consent from country officials before making the estimation files available through the UNAIDS website. A short form is required for informational purposes from which users are granted access to download the country-produced files. In 2014 and 2015 all but 6 countries agreed to share their country-developed national files; this number increased to 12 countries in 2016. Individuals requesting access to the country files are strongly encouraged to work with country estimates teams on analyses of the results.

### Evolution of the computational methods

HIV estimates are generated using software that includes a curve fitting model that is linked to a dynamic progression model. Major changes in methodology over the past 15 years have occurred in both the curve fitting model (the Estimation and Projection Package – EPP) and the progression model (the Spectrum/AIM module).

#### Changes in the curve fitting software

The software used for epidemic fitting has evolved steadily to address issues affecting the quality of the estimates as they were identified by the Reference Group and during national and global processes. The first set of UNAIDS global estimates prepared in 1997 used the Epimodel software developed originally by the Global Programme on AIDS to prepare projections by manually adjusting the model parameters to fit observed national trends. By 1999, UNAIDS had commissioned the development of an automatic fitting package based on the same gamma functions used in Epimodel; this formed the basis of the 1999 estimates. However, this generally led to fits with a sharp drop-off after the last data year and was incapable of producing the plateauing of prevalence that was apparent in many sub-Saharan African countries. In early 2001, the UNAIDS Reference Group held a meeting where several competing models were presented and the best features of each were taken to generate an epidemiologically motivated model that became known as the UNAIDS Reference Group model [19]. An early version of EPP was developed by the end of 2001 that allowed prevalence trends in urban/rural epidemics to be automatically fit by the Reference Group model. This software was used for generalized epidemics in the 2001 global estimates process, while a simpler method (UNAIDS Workbook), based on size estimates and measured prevalence in key populations, and a simple double logistic curve model for time trends was used for concentrated epidemics.

In 2002, EPP was further modified to allow users to define their national epidemic in terms of sub-national components, either geographic or sub-population (e.g. for key populations in concentrated epidemics). This version, along with UNAIDS Workbook, was applied in a series of regional workshops to prepare the global estimates in 2003 [34]. With continuous input from the Reference Group, EPP 2005, used for the 2005 global estimates, shifted to maximum likelihood fitting procedures, gave the user the ability to calibrate projections post-fitting, allowed for turnover in key populations, and introduced parameters allowing different prevalence levels for each surveillance site to address issues of spurious declines arising from the expansion of surveillance systems into lower-prevalence areas [35]. In EPP 2007, Bayesian methodologies were introduced to allow uncertainties to be calculated as part of the fitting process. In addition, calibration procedures were improved to make better use of national survey data, or in countries with no survey data, findings on the observed relationship of general population prevalence to prevalence among pregnant women attending antenatal clinics (ANC) [36,37].

By 2008, the Reference Group model was having difficulty fitting long-running epidemics in which behaviors had changed significantly; therefore, EPP 2009 added a new model, variable-R, which allowed for varying the force of infection over time [38]. In addition, incremental mixture importance sampling (IMIS) was added to the Bayesian fitting procedures to increase the number of resampled curves yielding better uncertainty estimates [39]. Acting on country feedback that the variable-R model fitting was too slow, two new models offering the same ability to vary the force of infection were introduced in EPP 2011: R-spline [40] and R-trend [41]. During this period the Weibull models for the HIV to AIDS and AIDS to death progression were replaced by a CD4 compartment model that allowed Spectrum and EPP to respond to changing national CD4 thresholds for ART eligibility [42]. From 2013, EPP was further improved to allow country teams to validate their results through a direct comparison of the modeled results with observed HIV and AIDS case reporting trends. Similarly, tools were added to allow comparison between one curve fit and a previous curve fit to improve the country teams’ understanding of the changes in the models.

The most recent updates to EPP have taken into account shifting ratios of prevalence in the general population to prevalence among pregnant women over time [43], improved the use of national survey data in the fitting procedures and switched to the Bayesian median of resampled curves instead of the mean to provide more stable fits in sparse data situations [44].

#### Changes in the Spectrum/AIM module

The Spectrum/AIM module uses prevalence and incidence trends as well as other demographic and epidemiological information to determine the other indicators of interest including the number of people living with HIV, new infections, AIDS deaths, need for ART and PMTCT, and the number of orphans. The first version was produced in 1997 and it was first used for global HIV estimates in 1999. Since 1999 there have been many improvements and additions. The demographic calculations were changed from five-year age groups to single years of age in 2001 and the inputs were based on the United Nations World Population Prospects estimates starting in 2003. Spectrum was linked to EPP prevalence trends in 2001 and in 2009 Spectrum started reading incidence trends from EPP.

The pattern of progression from infection to AIDS was initially based on a Weibull distribution with a median survival of 7, 9 or 11 years, then updated to 9 years from infection to AIDS death based on the Masaka cohort in 2003 [45], then updated to a median of 11 years’ survival based on data from the ALPHA network starting in 2007 [27]. In 2012 Spectrum implemented the CD4 compartment approach for adults where the HIV-positive population is tracked by CD4 count based on annual rates of CD4 count decline and mortality by CD4 category [42]. A similar approach was implemented for the pediatric model in 2015.

The effects of HIV infection on fertility were first implemented in 1999 and updated in 2009 based on a detailed analysis of Demographic and Health Survey data sets [46]. Mother-to child transmission was initially modeled as a single probability of transmission. The effects of antiretroviral prophylaxis were introduced in 2005 and new regimens have been added and were updated since then [47]. In the 2016 Spectrum/AIM module the transmission probability from mothers to their children was updated based on additional research; the most notable change was an important reduction in the transmission probability among women who seroconverted during the pregnancy [48].

The effects of ART were initially modeled as a three-year increase in median survival and then updated in 2012 with data from treatment cohorts provided by the IeDEA Consortium to include annual mortality on ART by age, sex, duration of treatment, region and CD4 count at treatment initiation [33]. Uncertainty ranges were first introduced in 1999 as fixed percentages based on the quality of surveillance data [49]. In 2005 a Monte Carlo procedure was added to calculate plausibility bounds on the basis of uncertainty around many of the key inputs [50]. In 2007 the uncertainty calculations began using prevalence draws from EPP [30]. Methods were introduced in 2009 to aggregate uncertainty to calculate bounds at regional and global levels.

In 2015 and 2016 new procedures were added to Spectrum to fit incidence trends based on case reports and vital registration of AIDS deaths. These tools are now widely used in countries with good case reporting systems and relatively complete reporting of AIDS deaths.

Similar to EPP, a number of tools were added to the Spectrum/AIM module to allow the country teams to validate their results, including the ability to compare the results to age-specific survey prevalence, ART coverage by age group, under-five all-cause mortality, all-cause adult mortality and prevalence among pregnant women.

### Impact of the process and computational adjustments on the HIV estimates

The changes to the methods over time have led to fluctuations in the estimated numbers of people living with HIV, new infections and AIDS-related deaths (Figure 1). While in 2001 the models available at the time estimated there were 40 million people living with HIV (no uncertainty bounds were avialble), the current models estimate that in 2001 there were 30 million (27.6–32.7 million) people living with HIV. The largest shift in the estimates occurred in 2007 with the systematic inclusion of household survey data in the models. The shift can be seen in the numbers of people living with HIV, new infections and AIDS-related deaths. Since 2007 the modeled global estimate of people living with HIV has stayed fairly constant with minor fluctuations from year to year, although between the 2015 and 2016 rounds, the estimated number of children living with HIV dropped by one third.

A number of publications have presented comparisons of the modeled estimates to empirical data. These comparisons allow UNAIDS and its Reference Group to refine the assumptions and methods for creating the estimates to adjust for quantifiable biases and limitations in available data [26,37,51–56]. Two articles on HIV incidence showed the similarities in the estimates to the empirical values, suggesting no changes were needed to the estimates’ values [52,55]. A more recent comparison of child estimates suggested the estimated numbers of children ages 10–14 were too low [], encouraging the reference group to consider reasons for the discrepancy. More recently a comparison of ART coverage estimates captured through surveys and a review of ART procurement data have substantiated the estimates of ART coverage [57].

Evidence of the impact of these changes has been seen in a number of countries. The dramatic change to the estimated number of people living with HIV in India due to the improved methodology is arguably the most noteworthy. From the 2006 round of estimates to the 2007 round of estimates the estimated number of people living with HIV in India changed from 5.7 million to 2.5 million [54]. The revised estimates led to an increased focus in India on the heterogeneity of the Indian HIV epidemic and its implications for geographic and key population focusing of responses to increase impact [58]. This also led to more focus in the response on key population-centered prevention saturation in high-prevalence areas [59].

## Lessons learned

A number of lessons can be taken from the challenges in the HIV estimates process, some of which have implications for producers of health-related statistics [14].

### Communicating results

One challenge is the confusion that occurs when the estimates fluctuate from year to year. Changes can occur as the methods are updated or as new surveillance or program data are used in the estimation models. These changes can be technically complex to explain to ministers of health, ministers of finance, journalists or other stakeholders. For example in 2015 UNAIDS reported there were 2.6 million children living with HIV globally. In 2016 the number was revised to 1.8 million, potentially causing confusion about whether 800,000 children living with HIV had died over the year [48]. To avoid confusion UNAIDS and partners explained the changes through multiple channels [60]. As research on the epidemic is published and as additional surveillance data become available, the improved estimates need to be released and carefully communicated to ensure a well-informed response.

The ownership of the estimates by country teams, which attend biennial regional trainings in estimation and projection that provide an opportunity to better understand changes in methodology and the impact of these changes on their own estimates, increases the influence of estimates on national decisions and positions them to better explain changes in the results.

### Timing of releasing estimates

A second challenge is the varying timelines of data availability and reporting needs in countries versus the need to release global data in a synchronized manner. Program and surveillance data are compiled in countries at different times of the year depending on the needs of national reporting, making it difficult to identify the best time of year to update the estimates with new data. UNAIDS and partners release reports on the HIV response around global HIV conferences or other big events. Although there is no easy solution for this challenge, a useful guiding principle is to release estimates as soon as they are finalized to maximize their usefulness and value for country processes.

### Learning from other models

The UNAIDS Reference Group invites groups creating alternative HIV models to its meetings to collaborate on ideas and share data. Models developed from a different perspective potentially offer new approaches and solutions for existing challenges. For example UNAIDS estimates have benefitted from the models recently developed by the Institute for Health Metrics and Evaluation (IHME). Although the IHME models build primarily on UNAIDS’ estimates with some modifications, their analyses on uncertainty around mortality estimates have been used in developing assumptions for Spectrum. Similarly the IHME results have been revised to more closely match the UNAIDS estimates [61]. The AIDS Epidemic Model (AEM) is another informative model taking a deterministic approach to estimate incidence, which can also inform policy and program decisions [62]. A number of countries in Asia have the data required for this model and prefer this approach. The Spectrum/AIM module can incorporate the AEM incidence estimates and produce the standard UNAIDS estimates. Models that are made specifically for a country, such as the Thembisa model from South Africa [63], provide more finely tuned country-specific estimates. These models also provide useful information and comparisons for the UNAIDS estimates [55]. Unfortunately, most countries do not have the required data and model parameters to produce such a model. UNAIDS compiles and reports results from standard models to ensure comparability of results between countries; however, the value of supporting and encouraging additional models for triangulation purposes is recognized.

### Models as a crutch

In some cases the reliance on models has left a gap in monitoring data. For example the UNAIDS estimates have been the primary impact measure for the Global Plan toward the elimination of new child infections and keeping their mothers alive [64]. By relying on models to estimate the impact of the PMTCT programs in the 21 high-burden priority Global Plan countries, these countries, technical partners and donors did not focus efforts on developing monitoring systems to follow children who were exposed to HIV to determine if they were infected and identify shortfalls in the program [65]. The PMTCT monitoring systems are not only critical for monitoring and reporting progress but also for linking children living with HIV to care and treatment providers. As Sustainable Development Goal targets are identified, countries and partners should ensure that any modeled data complement routine and empirical data and do not replace those data.

### Country ownership of estimates

The UNAIDS HIV estimates are only published if they are agreed to by senior country officials and UNAIDS technical experts. The data review and publication processes are important steps for both ensuring country ownership of the estimates and improving the use of the estimates as it ensures that senior country HIV program managers are reviewing the results.

Reasons for not publishing country estimates can be due to ongoing in-country analyses that are not completed by the time of the UNAIDS publication, or the use of alternative methods in the country. For example Ethiopia opted not to update its estimates in 2016 because it had a survey coming out later in the year and did not want to generate estimates twice in one year. Also in 2016 the Nigerian National AIDS Coordinating Authority asked UNAIDS to remove the Nigerian estimates on the UNAIDS website as questions arose in the country about some data generated by the surveillance system. The lack of data for these high-burden countries was problematic for partners such as the Global Fund to fight AIDS, Tuberculosis and Malaria and the US Government who use these estimates for their reporting on and planning of global investments.

In 2016, among the 193 United Nations countries or territories, no estimates were produced for 21 countries because they had populations of less than 250,000, while 12 countries did not produce estimates because they had too little data or were in a conflict situation. Estimates files were available for 160 countries and UNAIDS published estimates for 106 countries. Among the 54 countries for which no estimates were published, 39 were high-income countries with HIV surveillance data that are challenging to use in Spectrum. Estimates for the remaining 15 countries were either not finalized or agreed to by the time UNAIDS released the global estimates. All country estimates, regardless of whether they are published or not, are included in regional and global aggregates of key indicators.

The country review and signoff process has led to increased reflection on the data entered into the models and has subsequently improved the quality of surveillance systems over time and increased the likelihood of acting on it [49,66–68].

### Meeting GATHER criteria

In June 2016 the WHO launched an initiative to ensure the transparency, credibility and accurate interpretation of public health estimates. A set of 18 criteria were identified that must be met for a set of estimates to be compliant with the Guidelines for Accurate and Transparent Health Estimates Reporting (GATHER)  (http://gather-statement.org/). Table 1 summarizes the criteria and whether the UNAIDS HIV estimates have met them. Over the past 15 years the UNAIDSestimates have evolved from being unable to document most of the checklist items to meeting all but 5of the 18 criteria. The five criteria that are listed as ‘more detail required’ are difficult to summarize. For example, criterion 18 is ‘Discuss limitations of the estimates. Include a discussion of any modelling assumptions or data limitations that affect interpretation of the estimates.’ The HIV estimates have numerous limitations, starting with the fact that they are based on a large set of parameters, some of which are more or less representative of any specific country. Each country file will also have its own unique set of limitations regarding the data used in the model. While an overview of how the UNAIDS estimates meet the GATHER criteria is provided in Table 1, UNAIDS and its Reference Group are compiling a more thorough description of how the criteria are being met to publish on the Reference Group website (www.epidem.org).

**Table 1.** Evidence for meeting GATHER checklist for global health estimates.

GATHER checklist item (abbreviated)	Evidence of compliance	Status
Define indicators, population and time period of estimates	The estimates and their metadata are available from http://aidsinfo.unaids.org under the sections ‘People living with HIV’, ‘New HIV infections’, 'AIDS-related deaths', 'Treatment' and 'Elimination of mother to child transmission'.	Met
Funding sources	UNAIDS; US Government; Global Fund for AIDS, Malaria and Tuberculosis; Bill and Melinda Gates Foundation	Met
How data inputs were accessed	Data are compiled by country HIV estimates teams from program records and surveillance systems	Met
Inclusion and exclusion criteria	All survey and surveillance data are used in each country unless there are known data quality issues, such as, very short time series (surveillance data), or response rates are so low that findings are likely to be biased (national surveys). All available program data are used (number of ART and PMTCT patients) after validation by country teams and global partners	More detail required than can be provided here
Data sources and references, diagnostic methods, sample size	Prevalence and incidence trends are based on surveillance and survey data. Surveillance data include HIV prevalence measured at ante-natal clinics as well as surveillance conducted among key populations (sex workers, clients, men who have sex with men, people who inject drugs) and any additional groups relevant to a country’s epidemic for which data are available. Sample sizes are typically 300–500 per site. National surveys of HIV prevalence are available for some countries and usually include sample sizes of 5000–45,000. The sample sizes for each survey or study are included in the software. Testing is conducted according to standard HIV testing protocols with confirmatory tests for HIV positives	Met
Identify and describe any categories of input data that have potentially important biases	Numbers of people receiving antiretroviral medicines or therapy could be undercounted if not all sites have reported by the time of estimates development or might be double-counted if de-duplication efforts are not feasible or not conducted. Prevalence trends among pregnant women are not necessarily representative of the total population. Mortality data used to calibrate incidence curves might be under-reported due to stigma related to AIDS deaths. Survey measures of prevalence may be biased if refusal rates are high or if testing algorithms were not accurately implemented	More detail required than can be provided here
Additional data sources	Parameters for generalized epidemics are based primarily on demographic surveillance sites in eastern and southern Africa. Survival on ART is based on data from IeDEA consortium sites which might not be representative of all public providers	More detail required than can be provided here
All data inputs are available	Complete files with all input data can be downloaded from unaids.org. For those country files not publicly available, country team contact details can be requested from UNAIDS	Met
Conceptual overview of the methods	See Spectrum Quickstart guide and Spectrum manual at www.avenirhealth.org. Models are also fully described in published articles: [29–31,39–42,44,71]	Met
Description of the steps	Quickstart guide describing steps to create the estimate files is available from www.avenirhealth.org	Met
How were candidate models evaluated	See meeting reports at www.epidem.org.http://www.epidem.org/sites/default/files/reports/TechnicalRefinementsforSpectrum%202013.pdf	Met
Results of model evaluations and sensitivity analyses	See meeting reports at www.epidem.org.http://www.epidem.org/sites/default/files/reports/TechnicalRefinementsforSpectrum%202013.pdf	Met
Methods for calculating uncertainty	The following articles describe the methods for calculating uncertainty: [30,39]	Met
How to access code	Code can be requested from Avenir Health and East–West Center.	Being documented
Access to results	See http://aidsinfo.unaids.org	Met
Access to uncertainty results	See http://aidsinfo.unaids.org	Met
Interpretation of results in addition to other evidence and changes	See latest UNAIDS publications at www.unaids.org	Met
Limitations of the estimates	As of mid-2016 a selection of the limitations include: Incidence by age and sex is based on assumptions from cohort studies and might not reflect the situation in individual countries. For concentrated epidemics estimates of sizes of key populations may not match the population in which surveillance is conducted, making it difficult to estimate the total population of people living with HIV. In generalized epidemics information on prevalence among children is only available for a few countries against which to validate and compare the modelled estimates. Information on distribution of ART by age and sex is limited in most countries. Fertility patterns among women living with HIV (especially in concentrated epidemics) are not available, reducing the ability to accurately estimate children exposed to and potentially infected with HIV	More detail required than can be provided here

WHO has taken on a bold task in defining the GATHER criteria, especially given the variation in processes for generating health-related estimates. Documenting whether estimates meet these criteria will be equally difficult for other producers of health estimates.

### Future developments

In the coming years, UNAIDS and partners plan to move the software and the development of the estimates to the Internet, allowing faster calculations of estimates and more efficient support to countries during the development process. In addition a link through the Internet will allow country teams to pull the sub-national estimates (such as for provinces or districts) directly into their health information systems. An application has been developed for the District Health Information System (DHIS2) software to directly populate DHIS2 systems with the required denominators for key coverage variables calculated within Spectrum. Coming years will also see increasingly precise geospatial estimates at lower sub-national levels for which methods are currently under development [69,70]. Such estimates are needed for optimizing investments in the AIDS response by focusing them in the areas where the infection burden is highest, such as through geospatial models [69]. The increased data required for these geospatial estimates and for DHIS2 systems will make it increasingly useful to use cloud-based estimation platforms.

## Conclusions

The well-developed and well-documented UNAIDS HIV estimates process is the result of a number of factors. Country ownership of the estimates and user-friendly tools that allow teams to modify, update and validate their results have maintained country interest in the model. The numbers of countries participating in the workshops and submitting files increased from 131 in 2003 to 160 in 2016. The open cohort of leading scientists, implementers and researchers who guide the development of the models have ensured the quality of the results. In response to calls from program managers and donors for more frequent estimates, country teams have the capacity to produce estimates annually outside of workshop settings. The transparency and access to the files for researchers who want to further investigate the results or the methods have motivated improved methods. The value of the results is evident by the frequency of their use by global organizations such as the US Government, WHO, Global Fund to fight AIDS, Tuberculosis and Malaria, IHME and other partners. Finally the routine publications of the methods, data sources and results have led to UNAIDS meeting most of the GATHER criteria. While all modelled estimates have their limitations, the HIV estimates have evolved over time into valuable strategic information for countries and stakeholders to assess the HIV response.

## Paper context

Numerous articles have been written explaining specific changes to the HIV estimates methodology. However, no articles summarize the process and evolution of the HIV estimates. The increasing global reporting required for the Sustainable Development Goals, and potential increased reliance on estimations, make it important to take stock and document the challenges and lessons of generating global estimates. Country ownership, sound methodological guidance, good communication and transparency are key to successful estimates.
